# Immunomodulatory Effects of Canine Adipose Tissue Mesenchymal Stem Cell-Derived Extracellular Vesicles on Stimulated CD4^+^ T Cells Isolated from Peripheral Blood Mononuclear Cells

**DOI:** 10.1155/2021/2993043

**Published:** 2021-08-14

**Authors:** Takahiro Teshima, Yunosuke Yuchi, Ryohei Suzuki, Hirotaka Matsumoto, Hidekazu Koyama

**Affiliations:** ^1^Laboratory of Veterinary Internal Medicine, Department of Veterinary Clinical Medicine, School of Veterinary Medicine, Faculty of Veterinary Science, Nippon Veterinary and Life Science University, 1-7-1 Kyonan-cho, Musashino, Tokyo 180-8602, Japan; ^2^Research Center for Animal Life Science Nippon Veterinary and Life Science University, 1-7-1 Kyonan-cho, Musashino, Tokyo 180-8602, Japan

## Abstract

Adipose tissue-derived mesenchymal stem cells (ADSCs) have anti-inflammatory and immunomodulatory characteristics. Many studies have suggested that the immunomodulation of ADSCs is largely mediated by secreted paracrine factors. Various factors are secreted from ADSCs, among which extracellular vesicles are considered to play a major role in the communication between ADSCs and target cells. Several studies have reported the function of canine ADSC-derived extracellular vesicles (cADSC-EVs), but few studies have reported the immunomodulatory effects of cADSC-EVs on immune cells. The purpose of this study was to investigate the effects of cADSC-EVs on *in vitro*-stimulated CD4^+^ T cells isolated from peripheral blood mononuclear cells (PBMCs). cADSC-EVs were isolated from cADSCs under naive conditions or primed conditions by tumor necrosis factor-*α* (TNF*α*) and interferon-*γ* (IFN*γ*). The expression levels of several microRNAs in cADSC-EVs were altered by priming with TNF*α* and IFN*γ*. Culturing PBMCs stimulated with concanavalin A in the presence of naive or primed cADSC-EVs inhibited the differentiation of PBMCs and CD4^+^ T cells and promoted apoptosis of PBMCs. CD4^+^, CD8^+^, and CD4^+^CD8^+^ T cells were decreased, while CD3^+^CD4^−^CD8^−^ T cells were increased. T helper (Th) 1, Th2, Th17, and regulatory T (Treg) cells were analyzed by flow cytometry. cADSC-EVs inhibited the proliferation of Th1 and Th17 cells and enhanced Th2 and Treg cell proliferation. However, CD4^+^ T cells that had incorporated labeled cADSC-EVs comprised only a few percent of all cells. Therefore, these responses of stimulated CD4^+^ T cells may be due to not only direct effects of cADSC-EVs but also to indirect effects through interactions between cADSC-EVs and other immune cells. In conclusion, cADSC-EVs exert immunosuppressive effects on stimulated CD4^+^ T cells *in vitro*. These findings may be useful for further studies of immune diseases.

## 1. Introduction

Mesenchymal stem cells have various biological characteristics that include an immunomodulatory capacity [1–3]. Many studies have demonstrated that MSCs suppress the differentiation, proliferation, secretions, and migration of immune cells [4]. It has been documented that MSCs improve abnormal immune responses in autoimmune diseases *in vivo* and it has been thought that these benefits are partly due to secreted factors from MSCs [5–7]. Moreover, the MSC immunomodulatory ability is altered by inflammatory cytokine, such as those present in the inflammatory microenvironment. Stimulation with interferon-*γ* (IFN*γ*) enhances the immunosuppressive effects of MSCs. Priming MSCs with IFN*γ* upregulates indoleamine 2,3-dioxygenase (IDO), secretes important immunomodulatory molecules, such as prostaglandin E2 (PGE2), hepatocyte growth factor (HGF), transforming growth factor-*β* (TGF*β*), and chemokine ligand 2, and increases the expression of human leukocyte antigen class I and II molecules and costimulatory molecules [8]. Priming MSCs with tumor necrosis factor-*α* (TNF*α*) also promotes upregulation of immunoregulatory factors, such as PGE2, IDO, and HGF, but is much less pronounced compared with IFN*γ* priming [9]. However, the combination of inflammatory cytokines to stimulate MSCs may lead to additional effects. Priming MSCs with TNF*α* and IFN*γ* increase factor H production [10], which potently inhibits complement activation. Factor H secreted by MSCs is significantly suppressed by the inhibition of PGE2 and IDO. Therefore, priming MSCs with inflammatory cytokines is more useful for the treatment of immune-mediated diseases [11–13].

MSCs secrete large numbers of molecules, which include cytokines and growth factors, as well as extracellular vesicles (EVs), and these secretomes contribute to MSC abilities [14, 15]. Studies have focused on the immunomodulatory capacity of MSC secretomes for various types of immune cells and MSC-derived EVs (MSC-EVs) may have similar functions to parent cells in terms of immunomodulatory effects [4]. EVs transfer signaling molecules from one cell to another cell through a paracrine mechanism, because EVs enclose various active molecules, such as bioactive proteins, as well as lipids, mRNAs, and microRNAs [16], which contribute to regulating the gene expression and phenotypic transformation of target cells. MSC-EVs are incorporated by target cells through direct membrane fusion, receptor-mediated phagocytosis, and several other internalization mechanisms, which leads to subsequent activation of signal transduction pathways and involvement in various physiological and pathological processes that include immune responses [17–19]. Therefore, MSC-EVs are attracting attention as immunomodulators.

Similar to humans, immune-mediated diseases also exist in veterinary medicine, such as atopic dermatitis, inflammatory bowel disease, and immune-mediated arthritis. Almost all of these diseases have unclear mechanisms and some cases become intractable with current treatments. Therefore, MSC-based therapy is also expected to be an alternative treatment method for immune-mediated diseases [11–13]. However, few studies have documented the functions of canine ADSC- (cADSC-) EVs [20–22]. Furthermore, studies that focus on the effects of cADSV-EVs on T cells have not been reported. In this study, we investigated the immunomodulatory properties of cADSC-EVs on *in vitro*-stimulated CD4^+^ T cells isolated from peripheral blood mononuclear cells (PBMCs). Moreover, we evaluated whether immunomodulatory effects of cADSC-EVs on stimulated CD4^+^ T cells were enhanced by parent cells primed with inflammatory cytokines such as TNF*α* and IFN*γ*.

## 2. Materials and Methods

### 2.1. Stimulation of cADSCs with TNF*α* and IFN*γ*

cADSCs were isolated and used as described previously [23]. In brief, Adipose tissue was aseptically collected from falciform ligament fat of three anaesthetized dogs. The tissue was washed extensively in phosphate buffer solution (PBS), minced, and digested with collagenase type I (Sigma-Aldrich, Tokyo, Japan) at 37°C for 45 min with intermittent shaking. After washing with PBS and centrifuging, the pellets containing the stromal vascular fraction were resuspended, filtered through a 100-*μ*m nylon mesh, and incubated overnight in Dulbecco's Modified Eagle's medium (DMEM) supplemented with 10% fetal bovine serum (FBS) and a 1% antibiotic-antimycotic solution (Thermo Fisher Scientific, Tokyo, Japan) in a humidified atmosphere with 5% CO_2_ at 37°C. Unattached cells were removed by changing the medium, and the attached cells were washed twice with PBS. Thereafter, the medium was replaced every 3-4 days. At 80-90% confluence, the cells were detached with trypsin-EDTA solution (Sigma-Aldrich) and passaged repeatedly. The expression of several markers, such as CD14-FITC, CD29-PE, CD34-PE, CD44-PE, CD45-FITC, and CD90-PE, on these cells was determined by flow cytometry using a CytoFLEX (BECKMAN COULTER, Tokyo, Japan) [24]. cADSCs at passage 3 were seeded in 150-mm dishes (4 × 10^6^ cells/dish) and cultured in high glucose DMEM with 10% exosome-free FBS (Thermo Fisher Scientific) and a 1% antibiotic-antimycotic solution in a humidified atmosphere with 5% CO_2_ at 37°C. After 24 h, unattached cells were removed by changing the medium, and the attached cells were stimulated for 24 h with tumor necrosis factor-*α* (TNF*α*, 20 ng/ml) and interferon-*γ* (IFN*γ*, 20 ng/ml). Naive and primed cADSCs were cultured for 72 h.

### 2.2. cADSC-EV Isolation and Characterization

After 72 h of culture, the medium was harvested, and then, EVs were isolated by a MagCapture Exosome Isolation Kit PS (FUJIFILM Wako Pure Chemical, Osaka, Japan). In brief, the medium was centrifuged at 300 g for 5 min and then 1,200 g for 20 min at 4°C to remove cells and debris. The supernatant was added to 1/100 volumes of EV-Save Extracellular Vesicles Blocking Reagent (FUJIFILM Wako Pure Chemical, Osaka, Japan) and concentrated using an ultrafiltration unit (Vivaspin; SARTORIUS, Tokyo, Japan). After concentrating the supernatant, extracellular vesicles including exosomes were isolated in accordance with the manufacturer's instructions. Finally, the EVs were suspended in PBS.

The concentration and size of EVs were determined by nanoparticle tracking analysis (NTA) using a NanoSight LM10 (Malvern Panalytical, Tokyo, Japan) with the following parameters: camera level 12, threshold 8, 21.4°C, and five videos per analyzed sample. Visualization of EVs was assessed by transmission electron microscopy (H-7600; Hitachi High-Tech, Tokyo, Japan).

The total protein concentration of naive and primed EVs was measured by a BCA assay kit. Each 0.5 *μ*g protein sample in 20 *μ*l with 2.5 *μ*l NuPAGE LDS sample buffer was electrophoresed in 4%-12% Bis-Tris Gels and then transferred to a 0.45-*μ*m PVDF membrane. The membranes were blocked for nonspecific binding with Tris-buffered saline with 0.1% Tween-20 and 5% dry nonfat milk overnight at 4°C. After blocking, the membranes were incubated with a primary antibody for 1 h, and then with a secondary antibody for 1 h at room temperature. An anti-CD9 antibody (clone MM2/57; Bio-Rad, Tokyo, Japan) was used at a 1 : 200 dilution, an anti-CD63 antibody (clone H5C6; Novus Biologicals, CO, USA) was used at a 1 : 500 dilution, an anti-tumor susceptibility gene (TSG) 101 antibody (sc-7964; Santa Cruz BIOTECHNOLOGY, TX, USA) was used at a 1 : 200 dilution, and an HRP-conjugated anti-mouse IgG secondary antibody (sc-516102; Santa Cruz BIOTECHNOLOGY) was used at a 1 : 5000 dilution. Protein bands were visualized using a chemiluminescence detection kit. Images were captured using a CCD camera (ImageQuant LAS 500; Cytiva, Tokyo, Japan).

### 2.3. EV RNA Isolation and miRNA PCR Array

RNAs in naive and primed EVs were extracted using a Total Exosome RNA & Protein Isolation Kit (Thermo Fisher Scientific) and then concentrated using an RNA Clean-Up and Concentration Micro-Elute Kit (NORGEN BIOTEK, ON, Canada) in accordance with the manufacturers' instructions. Extracted miRNA quality and quantity were evaluated by a Qubit 4 fluorometer (Thermo Fisher Scientific). cDNA was synthesized from 10 ng miRNA in a 20 *μ*l reaction using a miScript II RT Kit (Qiagen, Tokyo, Japan) and then diluted in 180 *μ*l distilled water. The gene expression profile of canine miRNA was obtained by quantitative real-time PCR using a miScript miRNA PCR Array Dog miFinder (Qiagen). A PCR master mix was prepared using a miScript SYBR Green PCR Kit (Qiagen) in accordance with the manufacturers' instructions and added to a 96-well PCR array plate to be cycled as indicated. Data analyses were performed with free data analysis software (miScript miRNA PCR Array Data Analysis; Qiagen) using the *ΔΔ*CT method.

### 2.4. PBMC Isolation

Five healthy adult Beagles were used as blood donors. The dogs were handled in accordance with the animal care guidelines of the Institute of Laboratory Animal Resources, Nippon Veterinary and Life Science University, Japan. The Institutional Animal Care and Use Committee of Nippon Veterinary and Life Science University approved the experimental design (approval No. 2019-S58). Blood was collected from the jugular vein of each dog into heparinized tubes. PBMCs were immediately isolated by density gradient centrifugation using Histopaque-1077 and SepMate-15 (VERITAS, Tokyo, Japan). After isolation, PBMCs were resuspended in RPMI 1640 medium with 10% FBS, 1% antibiotic-antimycotic solution, 1% nonessential amino acid, 1% GlutaMAX (Thermo Fisher Scientific), and 50 *μ*M 2-mercaptoethanol.

### 2.5. Stimulation of PBMCs and Coculture with EVs

To determine the immunomodulatory effects of cADSC-derived EVs on stimulated PBMCs, 1 × 10^6^ PBMCs were seeded in a 12-well culture plate (1 ml per well). After 6 h culture, PBMCs were stimulated with 5 *μ*g/ml concanavalin A (ConA; Sigma-Aldrich) and cocultured with or without cADSC-derived EVs at various concentrations.

### 2.6. Cell Proliferation Assay

PBMCs were prelabeled with a 5 *μ*M carboxyfluorescein succinimidyl ester (CFSE) solution using a CFSE Cell Division Tracer Kit (BioLegend, Tokyo, Japan) before seeding and stimulation with ConA. PBMCs were cultured with naive or primed EVs at various concentrations (1, 5, and 10 *μ*g/ml). After 4 days, PBMCs were collected and washed with FACS buffer (PBS with 2% FBS). To inhibit nonspecific binding, canine Fc receptor binding inhibitor (Thermo Fisher Scientific) was added to cells, followed by incubation on ice for 20 min. After blocking, PBMCs were stained with anti-CD4-APC (clone: YKIX302.9, eBioscience, Tokyo, Japan) or the isotype control. The proliferation of PBMCs and CD4^+^ T cells among PBMCs was measured by flow cytometry.

### 2.7. Apoptosis Assay

PBMCs stimulated with ConA were cocultured with naive or primed EVs at various concentrations (1, 5, and 10 *μ*g/ml) for 3 days. Annexin V and propidium iodide (PI) staining was performed using a FITC Annexin V Apoptosis Detection Kit with PI (BioLegend) in accordance with the manufacturers' instructions. After staining, PBMC apoptosis was detected by flow cytometry.

### 2.8. CD3/CD4/CD8 T Cell Subset

To analyze the differentiation behavior of stimulated T cells, PBMCs stimulated with ConA were cocultured with naive or primed EVs at various concentrations (1, 5, and 10 *μ*g/ml) for 3 days. After coculture, PBMCs were collected, washed with FACS buffer, and incubated with canine Fc receptor binding inhibitor on ice for 20 min. Then, PBMCs were stained with anti-CD3-FITC (Clone: CA17.2A12, Bio-Rad), anti-CD4-RPE (Clone: YKIX302.9, Bio-Rad), and anti-CD8-Alexa Fluor 647 (Clone: YCATE55.9, Bio-Rad) or their respective isotype controls. Fluorescence was evaluated by flow cytometry.

### 2.9. Intracellular Cytokine Assay

To determine the proliferation behavior of T helper (Th) cells, PBMCs stimulated with ConA were cocultured with naive or primed EVs at various concentrations (1 or 3 *μ*g/ml) for 3 days. Then, PBMCs were stimulated with phorbol 12-myristate 13-acetate (PMA; 50 ng/ml, Sigma-Aldrich), and ionomycin (1 *μ*g/ml, Sigma-Aldrich) for 6 h and brefeldin A (10 *μ*g/ml, Sigma-Aldrich) for 4 h. After stimulation, PBMCs were collected, washed with FACS buffer, and incubated with canine Fc receptor binding inhibitor on ice for 20 min. PBMCs were stained with anti-CD3-FITC (Clone: CA17.2A12, Bio-Rad) and anti-CD4-APC (Clone: YKIX302.9, eBioscience, Tokyo, Japan) or their respective isotype controls. Then, PBMCs were fixed and permeabilized using Cyto-Fast Fix/Perm Buffer Set (BioLegend). Finally, PBMCs were stained with anti-IFN*γ*-RPE (clone: CC302, Bio-Rad), anti-IL-4-RPE (clone: CC303, Bio-Rad), and anti-IL-17A-RPE (clone: eBio64DEC17, Thermo Fisher Scientific) or their respective isotype controls. Analysis by flow cytometry was performed by measuring the frequency of IFN*γ*, IL-4, and IL-17A expression on gated CD3^+^CD4^+^ cells.

### 2.10. Regulatory T Cells

To analyze Treg cells, PBMCs stimulated with ConA were cocultured with naive or primed EVs at various concentrations (1 or 3 *μ*g/ml) for 3 days. After coculture, PBMCs were collected, washed with FACS buffer, and incubated with canine Fc receptor binding inhibitor on ice for 20 min. PBMCs were stained with anti-CD3-FITC (Clone: CA17.2A12, Bio-Rad), anti-CD4-APC (Clone: YKIX302.9, eBioscience), and anti-CD25-RPE (clone: P4A10, Thermo Fisher Scientific) or their respective isotype controls. Then, PBMCs were fixed and permeabilized using True-Nuclear Transcription Factor Buffer Set (BioLegend). Finally, PBMCs were stained with anti-Foxp3-eFluor450 (clone: FJK-16 s, Thermo Fisher Scientific) or that of isotype controls. Analysis by flow cytometry was performed by measuring the frequency of Foxp3 expression on gated CD3^+^CD4^+^CD25^+^ cells.

### 2.11. Uptake of EVs by CD4^+^ T Cells

cADSC-derived naive and primed EVs were stained with Vybrant DiI Cell-labeling Solution (Thermo Fisher Scientific), and then, unincorporated dye was removed from labeled EVs using an Exosome Spin Column (Thermo Fisher Scientific) in accordance with the manufacturers' instructions. PBMCs were stimulated with ConA (5 *μ*g/ml) for 3 days and then collected, washed with PBS, and resuspended in RPMI-1640 medium with 10% FBS, 1% antibiotic-antimycotic solution, 1% nonessential amino acid, 1% GlutaMAX, and 50 *μ*M 2-mercaptoethanol. One million stimulated PBMCs were cultured in a 12-well plate with 1 *μ*g/ml naive or primed labeled-EVs for 3 or 12 hours. Cells were fixed with 2% paraformaldehyde and then covered with a mounting medium with DAPI (VECTASHIELD: H-1200: VECTOR LABORATORIES, CA, USA). Immunofluorescence images were captured under a BZ-X700 multi-purpose microscope (KEYENCE, Osaka, Japan). The same samples without fixation were incubated with a canine Fc receptor binding inhibitor on ice for 20 min. Then, the cells were stained with anti-CD3-FITC (Clone: CA17.2A12, Bio-Rad) and anti-CD4-APC (Clone: YKIX302.9, eBioscience) or their respective isotype controls. Fluorescence was evaluated by flow cytometry.

### 2.12. Statistical Analysis

All data are presented as the mean ± standard deviation. Differences among multiple groups were assessed by one- or two-way analysis of variance and differences were compared using the Tukey-Kramer post hoc test. *P* < 0.05 was considered statistically significant. Statistical analyses were performed using Excel 2019 with add-in software Statcel 3.

## 3. Results

### 3.1. Characterization of cADSC-Derived EVs

**Figure 1:**
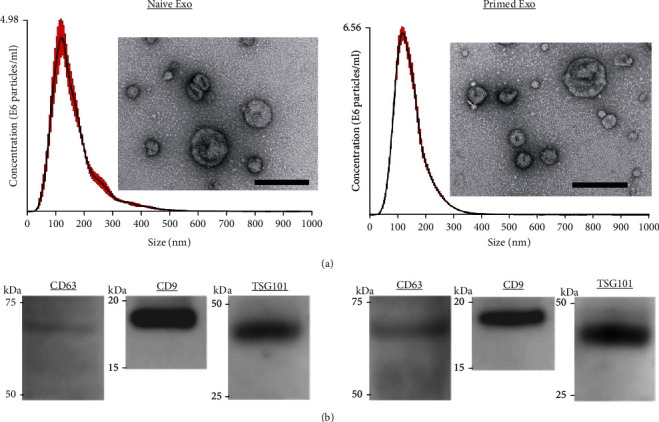
Characterization of cADSC-EVs. (a) Representative graph of nanoparticle tracking analysis and transmission electron microscopic images. Bar = 200 nm. (b) Immunoblots of cADSC-EVs for CD63 and CD9.

cADSCs were pretreated with or without TNF*α* and IFN*γ*, and then, an enriched fraction of EVs was collected from the supernatant using the PS affinity method. The size of isolated naive EVs was 166 ± 7.7 nm and that of primed EVs was 145 ± 1.5 nm. The concentrations of EVs were 34.4 ± 3.1 and 42.8 ± 1.9 × 10^9^ particles/ml for naive and primed EVs, respectively (Figure 1(a)). Both naive and primed EVs expressed specific exosomal markers CD9, CD63, and TSG101 [25]. The size of collected vesicles and marker expression indicated that cADSC-derived EVs isolated from the supernatant were an enriched fraction of exosomes (Figure 1(b)).

**Figure 2:**
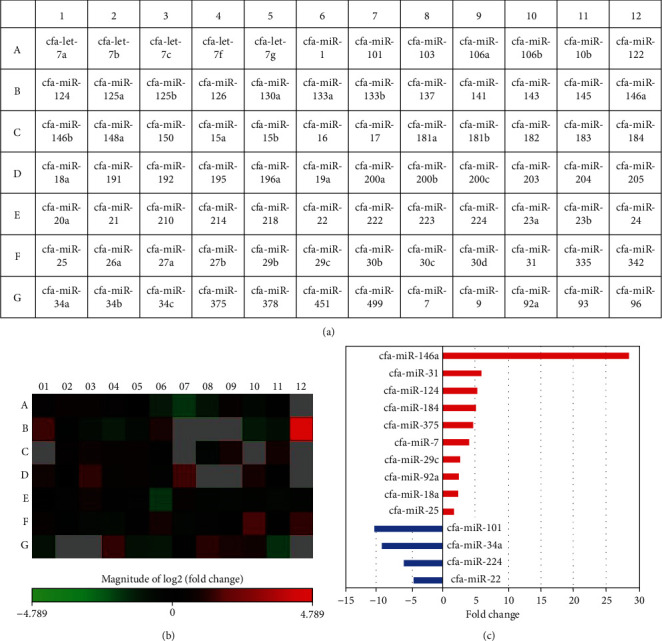
Differential expression of microRNAs in naive and primed cADSC-EVs. (a) Layout for Qiagen's canine miScript miRNA PCR Array. (b) Heatmap of microRNA expression between naive and primed cADSC-EVs. Red an increase and green/black indicate a decrease in relative expression in primed cADSC-EVs (n = 3 per group). Gray microRNAs represent not detected. (c) Significant differences in expression levels of microRNAs. Red bar indicates an increase in primed cADSC-EVs, and blue bar indicates an increase in naive cADSC-EVs.

Expression of exosome-associated microRNAs was investigated by a miRNA PCR array. Seventy microRNAs were detected in both naive and primed EVs, and expression levels of 14 microRNAs in primed EVs were significantly different compared with that in naive EVs (Figure 2). All data of fold regulation and *P* values of each microRNA are shown in Supplemental Material 1.

### 3.2. cADSC-Derived EVs Inhibit Proliferation of PBMCs and CD4^+^ T Cells

**Figure 3:**
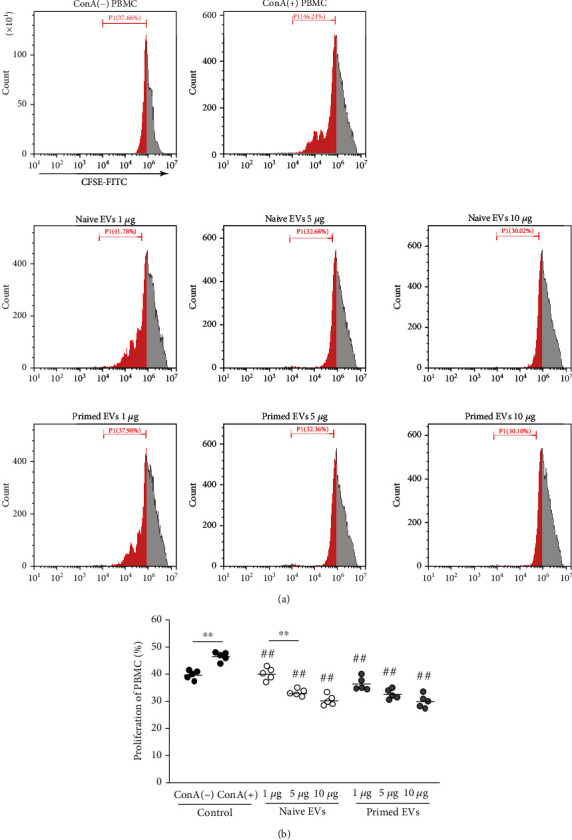
Proliferative ability of PBMCs cocultured with cADSC-EVs. (a) Proliferation of PBMCs was assayed using the CFSE method by flow cytometry. Segments represent the percentages of PBMC proliferation. (b) Comparison of proliferative PBMCs treated with various concentrations (1, 5, and 10 *μ*g/ml) of naive or primed cADSC-EVs. The inhibitive effects on PBMCs were not different between naive and primed EVs at the same concentrations. The solid line indicates the average. ^##^*P* < 0.01 vs. PBMCs stimulated with ConA. ^∗∗^*P* < 0.01, between groups.

**Figure 4:**
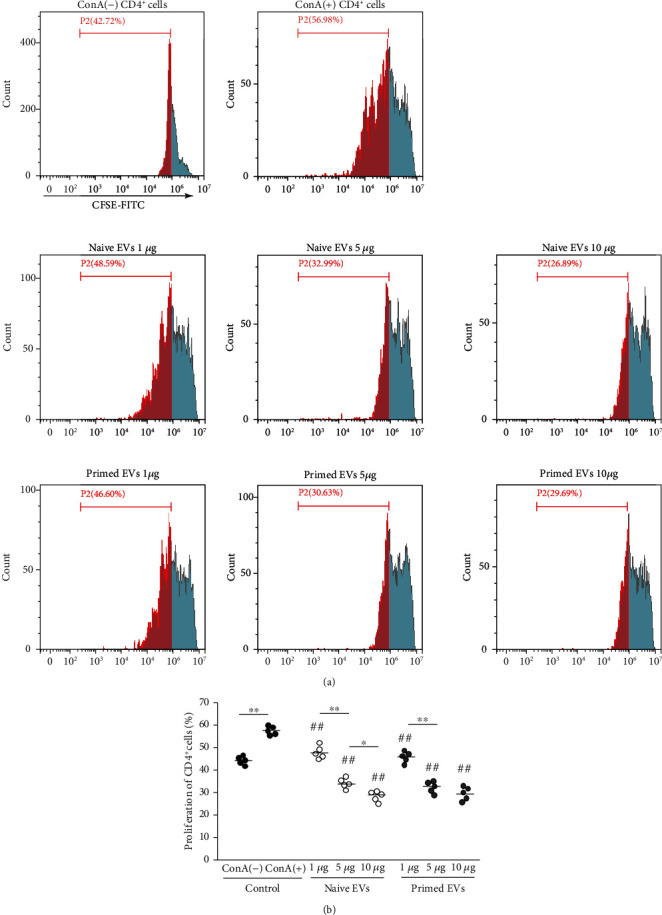
Proliferative ability of CD4^+^ T cells cocultured with cADSC-EVs. (a) Proliferation of CD4^+^ T cells was assayed using the CFSE method by flow cytometry. Segments represent the percentages of CD4^+^ T cell proliferation. (b) Comparison of proliferative CD4^+^ T cells treated with various concentrations (1, 5, and 10 *μ*g/ml) of naive or primed cADSC-EVs. The inhibitive effects on CD4^+^ T cells were not significantly different between naive and primed EVs at same concentrations. The solid line indicates the average. ^##^*P* < 0.01 vs. CD4^+^ T cells stimulated with ConA. ^∗^*P* < 0.05, between groups. ^∗∗^*P* < 0.01, between groups.

ConA (5 *μ*g/ml) stimulation for 4 days enhanced the proliferation of PBMCs and CD4^+^ T cells (unstimulated PBMCs: 40.0% ± 1.5%; ConA-stimulated PBMCs: 46.8% ± 1.5%; unstimulated CD4^+^ T cells: 43.8% ± 1.7%; ConA-stimulated CD4^+^ T cells: 57.0% ± 1.6%). The proliferation rate decreased gradually in both PBMCs (Figure 3) and CD4^+^ T cells (Figure 4) when PBMCs were cocultured in the presence of EVs. The inhibitive effects on PBMCs and CD4^+^ T cells were not different between naive and primed EVs at same concentrations.

### 3.3. cADSC-Derived EVs Induce Apoptosis of PBMCs

**Figure 5:**
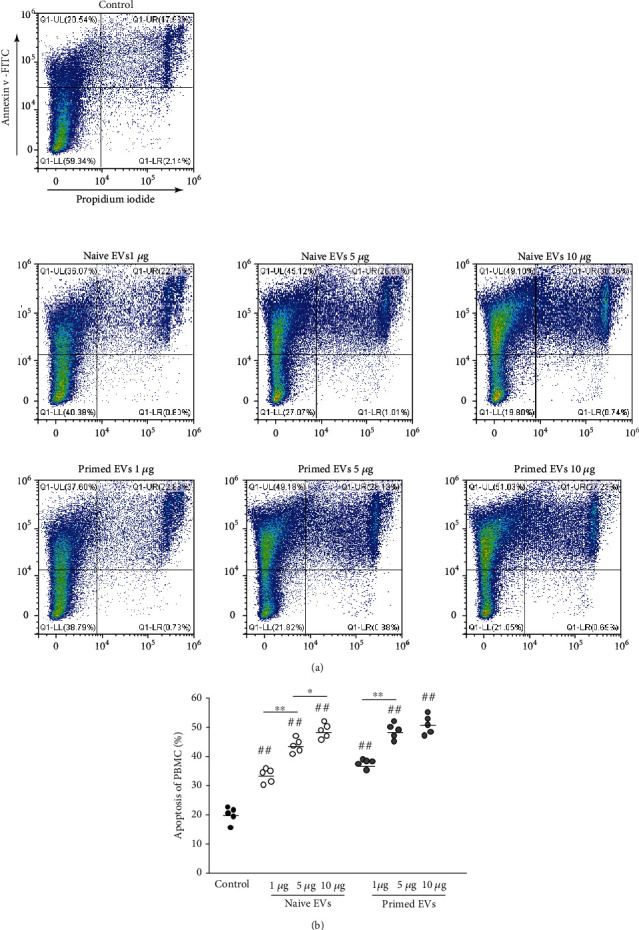
Apoptosis of PBMC cocultured with cADSC-EVs. (a) PBMCs stimulated with ConA were cultured in the presence of naive or primed cADSC-EVs at various concentrations (1, 5, and 10 *μ*g/ml) for 3 days. After culture, PBMCs were stained with Annexin V and PI and assayed by flow cytometry. (b) Comparison of apoptosis of PBMCs treated with various concentrations (1, 5, and 10 *μ*g/ml) of naive or primed cADSC-EVs. The induced ratio of PBMC apoptosis was not significantly different between naive and primed EVs at the same concentrations. The solid line indicates the average. ^##^*P* < 0.01 vs. PBMC stimulated with ConA. ^∗^*P* < 0.05, between groups. ^∗∗^*P* < 0.01, between groups.

When PBMCs were stimulated with ConA and cultured without cADSC-derived EVs, the apoptosis ratio was 20.0% ± 2.4%. However, PBMCs cocultured with various concentrations of naive and primed EVs had increased apoptosis (Figure 5). The induced ratio of PBMC apoptosis was not significantly different between naive and primed EVs at the same concentrations.

### 3.4. cADSC-Derived EVs Affect the T Cell Subset Distribution

**Figure 6:**
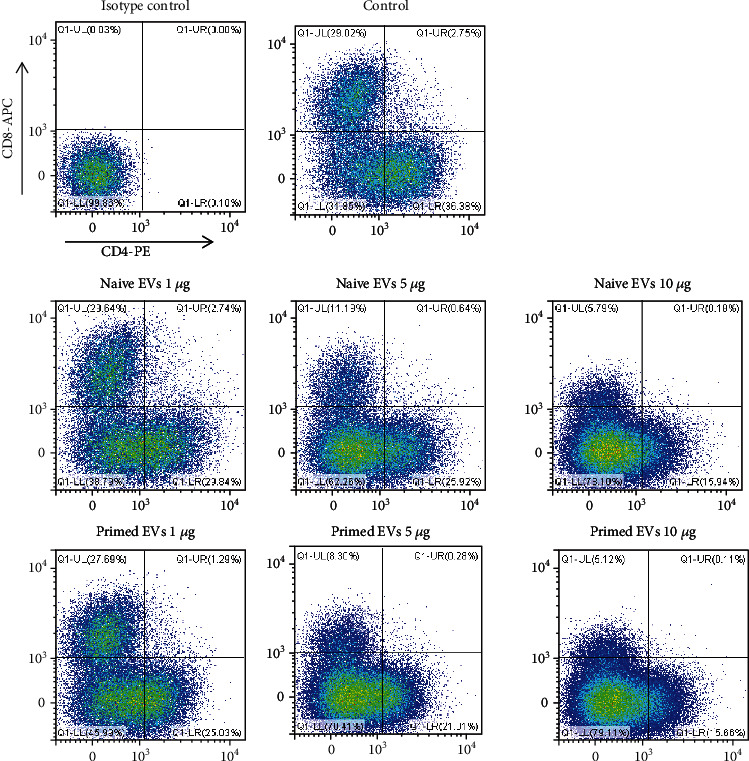
Immunophenotypic analysis of CD3, CD4, and CD8 in PBMCs cocultured with naive or primed cADSC-EVs at various concentrations (1, 5, and 10 *μ*g/ml).

After stimulating PBMCs with ConA for 3 days, the ratios of CD4^+^ T, CD8^+^ T, CD4^+^CD8^+^ T, and CD4^−^CD8^−^ T cells were 35.9% ± 2.4%, 29.1% ± 1.6%, 3.2% ± 0.5%, and 31.8% ± 4.0%, respectively. When PBMCs cocultured with EVs at various concentrations, the ratios of CD4^+^ T, CD8^+^ T, and CD4^+^CD8^+^ T cells were decreased proportionately, but that of CD4^−^CD8^−^ T cells was increased (Figure 6). The ratio of CD4^−^CD8^−^ T cells cultured with 5 and 10 *μ*g/ml primed EVs was significantly decrease compared with naive EVs (Table 1), but the T cell subset distribution was not different between naive and primed EVs at the same concentrations.

### 3.5. cADSC-Derived EVs Alter Differentiation into T Helper Cells

**Figure 7:**
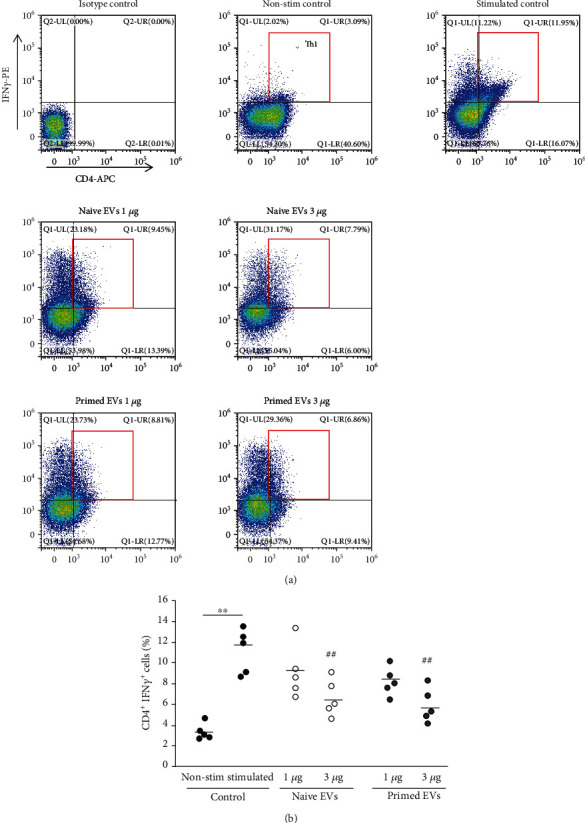
Differentiation into Th1 cells after coculture of PBMCs with cADSC-EVs. (a) Flow cytometric detection of CD4^+^IFN*γ*^+^ cells among CD3^+^ T cells (gated in red square). Non-stim control: PBMCs that were not stimulated with PMA and ionomycin. Stimulated control: PBMCs after coculture with cADSC-EVs and stimulated by PMA and ionomycin. (b) Comparison of CD4^+^IFN*γ*^+^ cells among CD3^+^ T cells after coculture of PBMCs with various concentrations (1 or 3 *μ*g/ml) of naive or primed cADSC-EVs. The ratio of Th1 cells was not significantly different between naive and primed EVs at the same concentrations. The solid line indicates the average. ^##^*P* < 0.01 vs. stimulated control. ^∗∗^*P* < 0.01, between groups.

**Figure 8:**
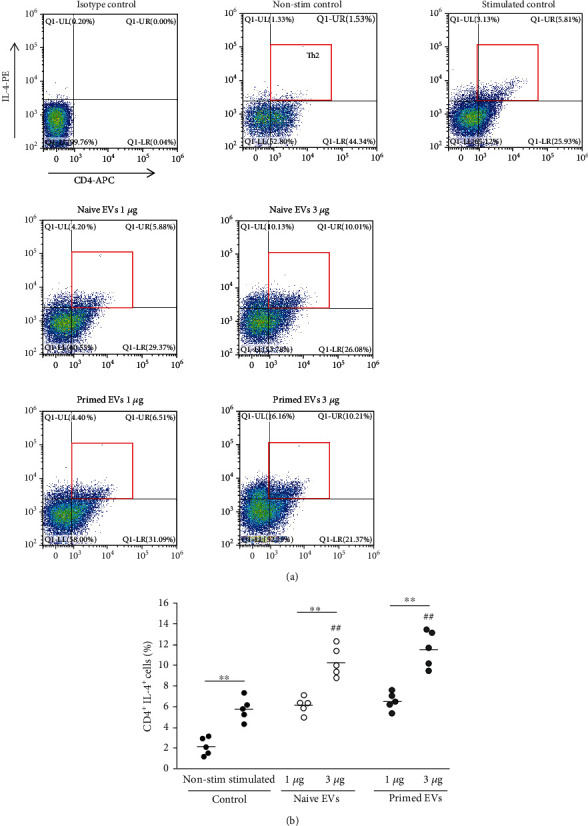
Differentiation into Th2 cells after coculture of PBMCs with cADSC-EVs. (a) Flow cytometric detection of CD4^+^IL-4^+^ cells among CD3^+^ T cells (gated in red square). Non-stim control: PBMCs that were not stimulated with PMA and ionomycin. Stimulated control: PBMCs after coculture with cADSC-EVs and stimulated by PMA and ionomycin. (b) Comparison of CD4^+^IL-4^+^ cells among CD3^+^ T cells after coculture of PBMCs with various concentrations (1 or 3 *μ*g/ml) of naive or primed cADSC-EVs. The ratio of Th2 cells was not different between naive and primed EVs at the same concentrations. The solid line indicates the average. ^##^*P* < 0.01 vs. stimulated control. ^∗∗^*P* < 0.01, between groups.

**Figure 9:**
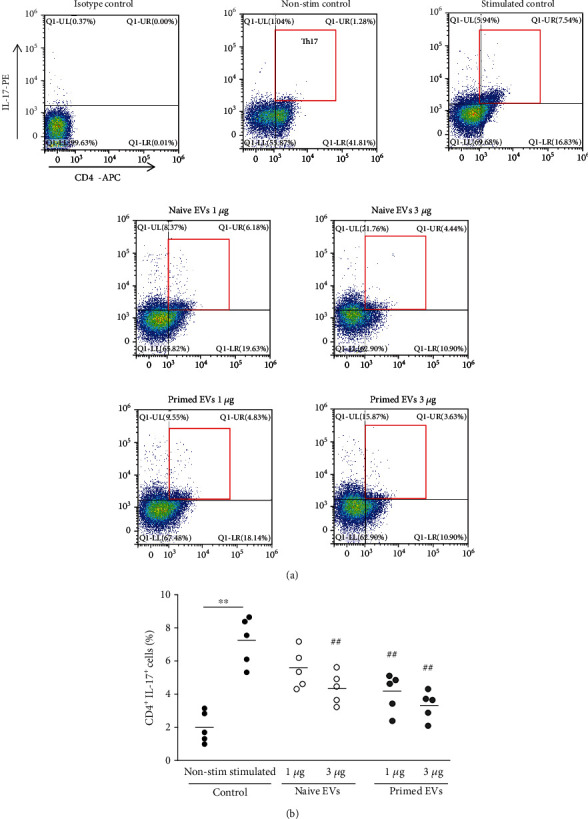
Differentiation into Th17 cells after coculture of PBMCs with cADSC-EVs. (a) Flow cytometric detection of CD4^+^IL-17^+^ cells among CD3^+^ T cells (gated in red square). Non-stim control: PBMCs that were not stimulated with PMA and ionomycin Stimulated control: PBMCs after coculture with cADSC-EVs and stimulated by PMA and ionomycin. (b) Comparison of CD4^+^IL-17^+^ cells among CD3^+^ T cells after coculture of PBMCs with various concentrations (1 or 3 *μ*g/ml) of naive or primed cADSC-EVs. The ratio of Th17 cells was not significantly different between naive and primed EVs at the same concentrations. The solid line indicates the average. ^##^*P* < 0.01 vs. stimulated control. ^∗∗^*P* < 0.01, between groups.

To examine Th1, Th2, and Th17 cells, intracellular cytokines IFNr, IL-4, and IL-17 were detected by flow cytometry. When PBMCs were stimulated with PMA and ionomycin for 6 h, intracellular expression of IFNr, IL-4, and IL-17 was increased compared with the unstimulated control. In the presence of EVs, the ratio of Th1 cells (CD4^+^IFNr^+^) among CD3^+^ T cells was decreased (3 *μ*g/ml naive EVs: 6.7% ± 1.6%; 3 *μ*g/ml primed EVs: 5.9% ± 1.5%) compared with no EVs (11.2% ± 2.0%) (Figure 7). Th2 cells (CD4^+^IL-4^+^) among CD3^+^ T cells were increased when PBMCs cocultured with EVs (3 *μ*g/ml naive EVs: 10.4% ± 1.3%; 3 *μ*g/ml primed EVs: 11.6% ± 1.6%) compared with no EVs (5.8% ± 1.0%) (Figure 8). Th17 cells (CD4^+^IL-17^+^) among CD3^+^ T cells after coculture with EVs were also suppressed (1 *μ*g/ml naive EVs: 5.5% ± 1.1%; 3 *μ*g/ml naive EVs: 4.4% ± 0.9%; 3 *μ*g/ml primed EVs: 3.3% ± 0.8%) compared with culture without EVs (7.2% ± 1.3%) (Figure 9). The ratio of Th1, Th2, and Th17 cells was not different between naive and primed EVs at the same concentrations.

### 3.6. cADSC-Derived EVs Enhance Treg Cells

**Figure 10:**
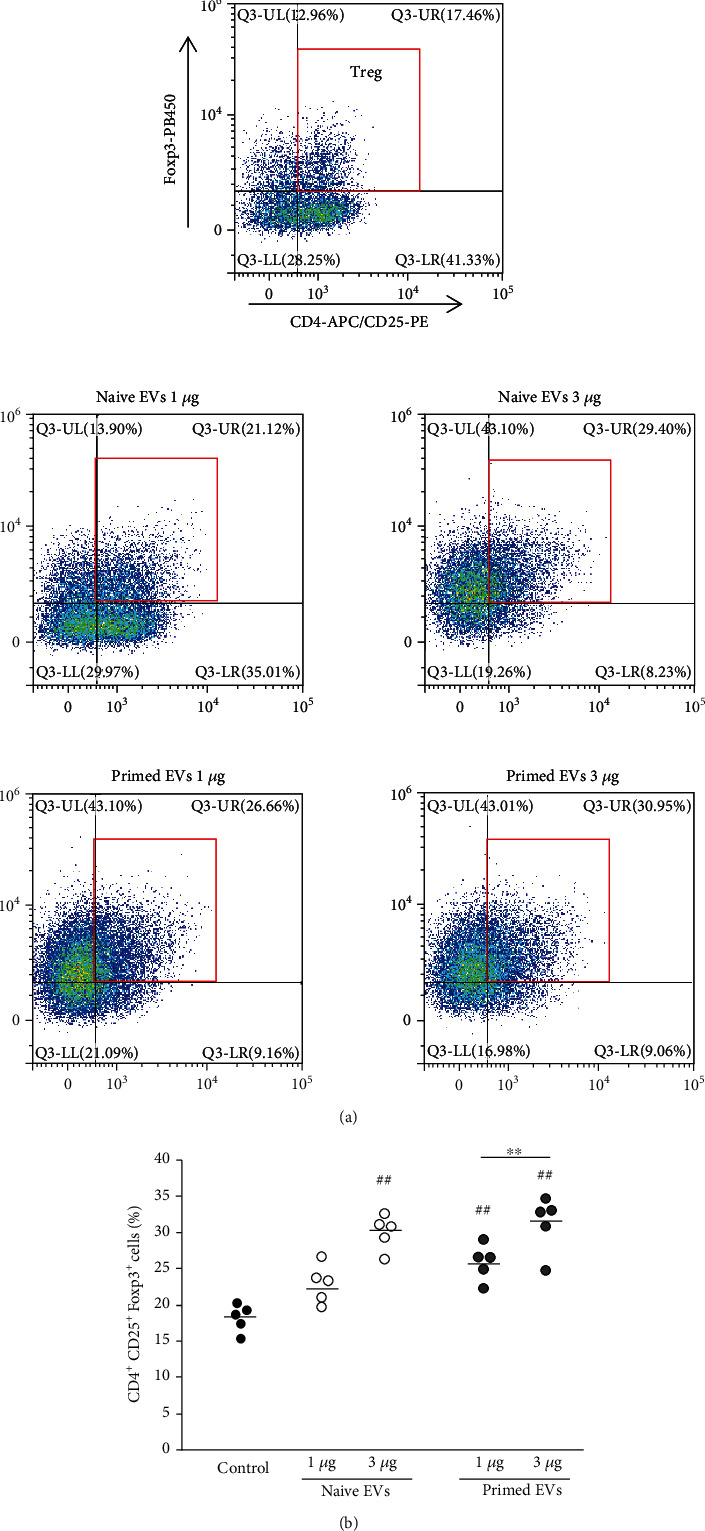
Treg cells after coculture of PBMCs with cADSC-EVs. (a) Flow cytometric detection of CD3^+^CD4^+^CD25^+^Foxp3^+^ cells (gated in red square). Control: PBMCs cultured without cADSC-EVs. (b) Comparison of the population of CD3^+^CD4^+^CD25^+^Foxp3^+^ cells after coculture of PBMCs with various concentrations (1 or 3 *μ*g/ml) of naive or primed cADSC-EVs. The ratio of Treg cells was not significantly different between naive and primed EVs at the same concentrations. The solid line indicates the average. ^##^*P* < 0.01 vs. control. ^∗∗^*P* < 0.01, between concentrations of 1 and 3 *μ*g/ml.

The ratio of Treg (CD3^+^CD4^+^CD25^+^Foxp3^+^) cells among PBMCs stimulated with ConA was 18.2% ± 1.7%. When PBMCs were cultured with EVs, the ratio of Treg cells in CD4^+^ T cells was elevated significantly (1 *μ*g/ml naive EVs: 23.0% ± 2.4%; 3 *μ*g/ml naive EVs: 30.1% ± 2.1%; 1 *μ*g/ml primed EVs: 26.0% ± 2.2%; 3 *μ*g/ml primed EVs: 31.3% ± 3.5%) (Figure 10). The ratio of Treg cells was not different between naive and primed EVs at the same concentrations.

### 3.7. cADSC-Derived EV Uptake by CD4^+^ T Cells

**Figure 11:**
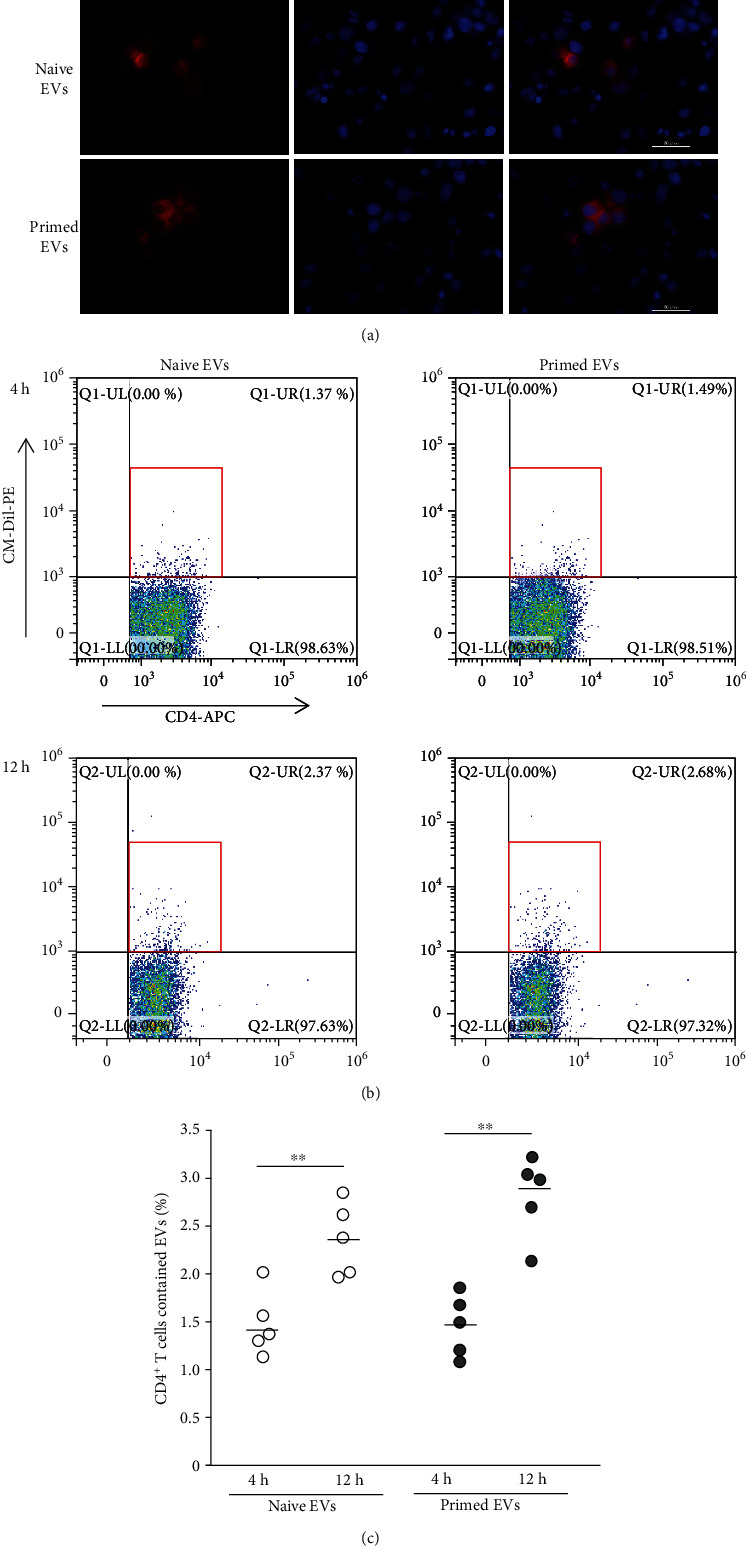
Incorporation of cADSC-EVs into stimulated T cells. (a) Immunofluorescence staining of labeled EVs and T cells stimulated with ConA. cADSC-EVs were labeled by Vybrant DiI (red) and nuclei were stained by DAPI (blue). (b) Flow cytometric detection of cADSC-EVs incorporated into CD4^+^ T cells (gated in red square). (c) Comparison of the population of CD4^+^ T cells that contained cADSC-EVs at 4 and 12 hours after coculture of stimulated PBMCs with 1 *μ*g/ml cADSC-EVs. The solid line indicates the average. ^∗∗^*P* < 0.01, between 4 and 12 h.

T cell-enriched PBMCs were coculture with cADSC-derived EVs for 3 or 12 hours. Both naive and primed EVs were incorporated into T cells and CD4^+^ T cells treated with EVs were observed by flow cytometry (Figure 11). The ratio of CD4^+^ T cells with cADSC-derived EVs at 12 h was significantly elevated in both naive and primed EVs compared to those at 3 h.

## 4. Discussion

Mesenchymal stem cells (MSCs) have immunomodulatory and anti-inflammatory abilities by secreting numerous factors such as extracellular vesicles, cytokines, chemokines, and growth factors. As research on MSC-based therapies has proceeded, exosomes have been focused on as important factors that exert immunomodulatory effects. Exosomes are typically small membrane vesicles (30-150 nm) that include extracellular vesicles (30-1000 nm) secreted by various cell types. The classically used protocol to isolate exosomes is ultracentrifugation, but there are currently several exosome isolation methods based on different principles [26, 27]. The ultracentrifugation method is based on the principles of precipitation and sedimentation. Therefore, ultracentrifugation also concentrates nonspecifically sedimentable particles, which include non-EV proteins, and is associated with low reproducibility because of the loss of unstable and invisible pellets after centrifugation. Conversely, the PS affinity method is based on the high affinity of T cell immunoglobulin domain and mucin domain-containing protein 4 that strongly binds to phosphatidylserine expressed on the surface of EVs. The PS affinity method only requires general laboratory equipment. Moreover, this method isolates EVs with higher quality and reproducibility compared with ultracentrifugation [28]. In this study, we collected cADSC-EVs using the PS affinity method. The results of NTA and analyses of exosome markers (CD9, CD63, and TSG101) suggested that the cADSC-EVs isolated from culture supernatants were an enriched exosome fraction.

In this study, we examined the microRNA expression profile of naive and TNF*α*/IFN*γ*-primed cADSC-EVs. microRNAs, which are a class of noncoding RNAs of 20-22 base pairs, regulate the expression of multiple RNAs and play an important role in various biological processes that include the immune response. Several microRNAs affect the immune response in T cell development, proliferation, differentiation, and function [29, 30]. The microRNA profiles showed that several microRNAs in cADSC-EVs varied after stimulation with TNF*α* and IFN*γ*. For example, miR-146a, which was overexpressed in primed cADSC-EVs, acts as a negative regulator of T cells and promotes Treg cell functions [30]. MiR-34a, which was downregulated in primed cADSC-EVs, plays an important role in T cell activation by targeting >30 genes across different cellular pathways that control the immune response [31].

Several studies have reported that primed EVs increase immunomodulatory functions compared with naive EVs [32, 33], whereas other reports have indicated that primed EVs do not enhance the immunomodulatory effects of MSC-EVs [34, 35]. In our study, primed cADSC-EVs did not enhance any functions of PBMCs or stimulated CD4^+^ T cells. The reason that the immunomodulatory functions of primed cADSC-EVs were not enhanced remains unclear, but several hypotheses were proposed. First, packaged soluble factors inside EVs may be related [34]. The properties of factors secreted from MSCs change depending on MSC culture conditions [5]. There is evidence suggesting that immunomodulatory effects of MSCs are enhanced in response to inflammatory stimulation [5]. Inflammatory stimuli by TNF*α* and IFN*γ* have been commonly used to research the immune and regenerative functions of MSCs and are known to enhance the immunosuppressive properties of MSCs [6]. For example, immunomodulatory effects of T cells are attributed to the upregulation of IDO. MSC-EVs also packaged various active proteins, which included IDO, but previous studies have demonstrated no significant change of IDO in PBMCs cocultured with or without MSC-EVs and thus suggested that the mechanism that underlies the immunomodulatory capacities of MSC-EVs is different from that of MSC soluble factors [36]. Second, stimulation methods of T cells may be related [35]. In this study, PBMCs were stimulated with ConA. ConA is an antigen-independent mitogen and is widely used as a T cell stimulus. However, T cell activation by an anti-CD3/28 antibody can be used in humans and mice as an antigen-dependent method. Antigen-presenting cells partially mediate T cell suppression induced by MSCs [37]. Therefore, another explanation might be a lack or small number of antigen-presenting cells in our experimental setting. In our study, we analyzed characteristic features by only comparing the expression of microRNAs between naive and primed cADSC-EVs, but there are many other components such as proteins and mRNAs. Furthermore, several cytokines and growth factors have been reported in MSCs, such as TGF*β*, TNF*α*, IFN*γ*, IL-4, and IL-10 [6]. Further studies are required to clarify the alteration of components in cADSC-EVs with or without priming and using other stimulation methods.

MSCs suppress T cell proliferation [38], B cell activity [39], and NK cell proliferation [40] and interfere with the differentiation, maturation, and function of dendritic cells [41]. The purpose of this study was to investigate the immunomodulatory capacity of cADSC-EVs for T cells. In this study, PBMCs were stimulated with ConA. The immunomodulatory effects exerted by MSC-EVs on activated T cells remain a widely discussed topic. Primed and naive human MSC-EVs cocultured with PBMCs suppress the proliferation of T cells but do not affect that of B and NK cells [42]. Other studies have shown that MSC-EVs inhibit the proliferation of NK and B cells, but their effects on the proliferation of T cells remain unclear [32, 43]. Our results demonstrated that cADSC-EVs suppressed the proliferation of PBMCs and CD4^+^ T cells and enhanced apoptosis of PBMCs. It has been demonstrated that MSC-EVs carry various active molecules that may contribute to the MSC-EV capacity to inhibit T cell proliferation and activation and induce T cell apoptosis [4]. However, the mechanisms of suppressive proliferation and induced apoptosis in T cells by MSC-EVs remain unclear. One report has demonstrated that TNF*α*/NF-*κ*B signaling in MSCs is required to inhibit T cell proliferation [44]. Therefore, MSC-EVs effects may also be related to activation of NF-*κ*B. Another recent study has demonstrated that MSC-EVs suppress the proliferation of T cells by inducing cell cycle arrest through p27kip1/Cdk2 signaling [45]. P27kip1 is a representative factor that induces cell cycle arrest by downregulating Cdks, and MSC-EVs were associated with upregulation of p27kip1 and downregulation of Cdk2. It has been demonstrated that MSCs induce apoptosis of activated T cells through the FAS ligand-dependent FAS signaling pathway but do not induce naive T cell apoptosis *in vitro* [46]. However, there are no reports of a correlation between MSC-EVs and the FAS signaling pathway. The only report of the mechanism of T cell apoptosis induction by MSC-EVs assumed that MSC-EVs induce T cell apoptosis possibly through an MSC-EV mediated mechanism via the adenosine A2A receptor pathway [47]. After coculture of PBMCs stimulated with ConA in the presence of cADSC-EVs, CD4^+^, CD8^+^, and CD4^+^CD8^+^ T cells were decreased, whereas CD3^+^CD4^−^CD8^−^ T cells were increased. A study has evaluated the status of CD4^+^ and CD8^+^ T cells by categorizing in accordance with CD45RA and CCR7 expression, such as naive (CD45RA^+^CCR7^+^), central memory (CD45RA^−^CCR7^+^), effector memory (CD45RA^−^CCR7^−^), and terminally differentiated effector memory cells (CD45RA^+^CCR7^−^) [48]. This study demonstrated that human ADSC-EVs inhibited the differentiation of CD4^+^ and CD8^+^ T cells into terminally differentiated effector memory cells. It is unclear how cADSC-EVs inhibited the differentiation of CD4^+^ and CD8^+^ T cells, but we hypothesize that cADSC-EVs may suppress T cell differentiation toward immature phenotypes. A study has examined the effects of MSC-EVs on acute graft-versus-host disease and found that MSC-EVs are associated with the preservation of circulating naive T cells because of the unique microRNA profiles of MSC-EVs [49]. microRNAs are also thought to be related to changes in the proliferation, apoptosis, and distribution of T cells induced by cADSC-EVs.

To clarify the alteration of the immune response of Th cells induced by cADSC-EVs, intracellular cytokines were detected by flow cytometry. Both naive and primed cADSC-EVs increased the ratio of Th2 cells but decreased Th1 cells. Some studies of autoimmune diseases have reported that MSC-EVs drive a shift from Th1 toward Th2 cells and rebalances Th1/Th2 cells by downregulating proinflammatory cytokines TNF*α* and IFN*γ* and upregulating anti-inflammatory cytokines IL-10 or IL-4 [36, 50]. Moreover, cADSC-EVs inhibited activated T cell differentiation into Th17 cells and promoted differentiation into Treg cells. Such regulation has also been observed in human MSC-EVs [4], but the mechanism is unclear. On the basis of the immune balance effect exerted by cADSC-EVs on Th and Treg cells, our results indicate that cADSC-EVs may act as an ameliorating agent for autoimmune diseases.

In this study, we examined the effects of cADSC-EVs on T cells and focused on CD4^+^ T cells isolated from PBMCs. cADSC-EVs affected CD4^+^ T cell proliferation, apoptosis, and differentiation, but CD4^+^ T cells that took up cADSC-EVs were only a few percent of all cells. This result was similar to previous studies of unfractionated PBMCs or purified T cells, and MSC-EVs were almost entirely incorporated by monocytes [32, 51]. MSC-EVs also act on monocytes/macrophages [4]. Thus, the influence of T cells was not only a direct effect of cADSC-EVs but also included indirect effects of monocytes affected by cADSC-EVs. Further studies of the interactions between cADSC-EVs, T cells, and monocytes are needed to clarify the effects of cADSC-EVs on the immune response.

## 5. Conclusions

Our study shows that cADSC-EVs have an immunoregulatory function by inducing PBMC apoptosis, suppressing the proliferation of PBMCs and stimulated CD4^+^ T cells as well as the differentiation of CD4^+^ and CD8^+^ T cells, and changing Th1/Th2/Treg cell populations *in vitro*. To evaluate whether cADSC-EVs have beneficial effects on immune diseases and are practical for use in treatments of immune diseases, further study is needed to analyze which components of cADSC-EVs exert the individual immunosuppressive effect and the pathways by which these immunosuppressive effects occur.

## Figures and Tables

**Table 1:** Comparison of CD4^+^, CD8^+^, CD4^+^CD8^+^, and CD4^−^CD8^−^ T cell populations after coculture of PBMCs with cADSC-EVs.

	Control	Naive EVs			Primed EVs		
		1 *μ*g/ml	5 *μ*g/ml	10 *μ*g/ml	1 *μ*g/ml	5 *μ*g/ml	10 *μ*g/ml
CD4^+^	35.9 ± 2.4	29.4 ± 1.9^∗∗^	26.1 ± 1.7^∗∗^	15.3 ± 1.9^∗∗^	25.7 ± 2.2^∗∗^	21.7 ± 1.7^∗∗^	15.5 ± 2.0^∗∗^
CD8^+^	29.1 ± 1.6	29.3 ± 2.0	11.8 ± 1.8^∗∗^	5.7 ± 1.3^∗∗^	27.1 ± 1.8	8.3 ± 1.6^∗∗^	5.4 ± 1.6^∗∗^
CD4^+^CD8^+^	3.2 ± 0.5	3.0 ± 0.8	1.0 ± 0.5^∗∗^	0.2 ± 0.1^∗∗^	1.4 ± 0.5^∗∗^	0.3 ± 0.1^∗∗^	0.2 ± 0.1^∗∗^
CD4^−^CD8^−^	31.8 ± 4.0	38.3 ± 2.2^∗^	61.1 ± 3.2^∗∗^	78.8 ± 1.3^∗∗^	45.7 ± 3.2^∗^	69.7 ± 1.7^∗∗^	78.9 ± 2.8^∗∗^

Data are shown as the mean ± S.D. The ratio of T cell subset was not significantly different between naive and primed EVs at the same concentrations. ^∗∗^*P* < 0.01 vs. control (PBMCs that were not cocultured with cADSC-EVs). ^∗^*P* < 0.05 vs. control.

## Data Availability

The data used to support the findings of this study are available from the corresponding authors upon reasonable request.

## Supplementary Materials

Supplementary Materials Supplementary Material 1: comparison of the microRNAs expression between naive and primed cADSC-EVs.
