# Supplementary material for: Immunomodulatory Effects of Canine Adipose Tissue Mesenchymal Stem Cell-Derived Extracellular Vesicles on Stimulated CD4+ T Cells Isolated from Peripheral Blood Mononuclear Cells
Source: J Immunol Res. 2021 Aug 14;2021:2993043. doi: 10.1155/2021/2993043 (PMC8384509; doi:10.1155/2021/2993043)
Supplement: Supplementary Materials — Supplementary Material 1: comparison of the microRNAs expression between naive and primed cADSC-EVs. [file 2993043.f1.pdf]

**Position**	**Mature ID**	**Fold Regulation** (comparing to naive cADSC-EVs)	**p-value**
A01	cfa-let-7a	1.3241	0.335496
A02	cfa-let-7b	1.8241	0.149568
A03	cfa-let-7c	1.7773	0.079315
A04	cfa-let-7f	1.3157	0.201034
A05	cfa-let-7g	1.0295	0.82897
A06	cfa-miR-1	-2.6833	0.190422
A07	cfa-miR-101	-10.3404	0.019085
A08	cfa-miR-103	-4.0471	0.095043
A09	cfa-miR-106a	1.757	0.114489
A10	cfa-miR-106b	-1.9776	0.42919
A11	cfa-miR-10b	-1.3457	0.834631
A12	cfa-miR-122	Not detected	N/A
B01	cfa-miR-124	5.3816	0.000428
B02	cfa-miR-125a	-1.081	0.669637
B03	cfa-miR-125b	-1.8087	0.307481
B04	cfa-miR-126	-2.7014	0.57625
B05	cfa-miR-130a	-2.7106	0.134387
B06	cfa-miR-133a	1.1755	0.991237
B07	cfa-miR-133b	Not detected	N/A
B08	cfa-miR-137	Not detected	N/A
B09	cfa-miR-141	Not detected	N/A
B10	cfa-miR-143	-2.9104	0.264676
B11	cfa-miR-145	-2.2233	0.580382
B12	cfa-miR-146a	28.5491	0.000014
C01	cfa-miR-146b	Not detected	N/A
C02	cfa-miR-148a	1.3264	0.399308
C03	cfa-miR-150	-1.181	0.431676
C04	cfa-miR-15a	1.6998	0.187191
C05	cfa-miR-15b	1.7914	0.19375
C06	cfa-miR-16	1.2769	0.328601
C07	cfa-miR-17	Not detected	N/A
C08	cfa-miR-181a	-1.4544	0.417074
C09	cfa-miR-181b	2.726	0.133054
C10	cfa-miR-182	Not detected	N/A
C11	cfa-miR-183	1.9656	0.549292
C12	cfa-miR-184	Not detected	N/A
D01	cfa-miR-18a	2.3675	0.029935
D02	cfa-miR-191	-1.0293	0.884403
D03	cfa-miR-192	2.7809	0.393788
D04	cfa-miR-195	1.5466	0.145785
D05	cfa-miR-196a	2.7462	0.834191
D06	cfa-miR-19a	-1.2498	0.827176
D07	cfa-miR-200a	4.2954	0.058773
D08	cfa-miR-200b	Not detected	N/A

D09	cfa-miR-200c	Not detected	N/A
D10	cfa-miR-203	1.1487	0.933257
D11	cfa-miR-204	-1.3852	0.508459
D12	cfa-miR-205	Not detected	N/A
E01	cfa-miR-20a	1.0335	0.696267
E02	cfa-miR-21	-1.1763	0.712576
E03	cfa-miR-210	2.1925	0.126636
E04	cfa-miR-214	1.2058	0.264335
E05	cfa-miR-218	-1.8623	0.102677
E06	cfa-miR-22	-4.3417	0.039414
E07	cfa-miR-222	1.655	0.052281
E08	cfa-miR-223	-1.3931	0.669417
E09	cfa-miR-224	-5.8538	0.020921
E10	cfa-miR-23a	-1.2483	0.913658
E11	cfa-miR-23b	-1.5461	0.293023
E12	cfa-miR-24	-1.257	0.98307
F01	cfa-miR-25	1.7486	0.029327
F02	cfa-miR-26a	-1.0872	0.89464
F03	cfa-miR-27a	-1.5751	0.470253
F04	cfa-miR-27b	-1.7898	0.104568
F05	cfa-miR-29b	1.2648	0.997484
F06	cfa-miR-29c	2.7495	0.033696
F07	cfa-miR-30b	-1.4664	0.064719
F08	cfa-miR-30c	-1.186	0.840265
F09	cfa-miR-30d	1.1475	0.958333
F10	cfa-miR-31	6.0386	0.000085
F11	cfa-miR-335	1.1397	0.559986
F12	cfa-miR-342	3.579	0.127786
G01	cfa-miR-34a	-9.197	0.000002
G02	cfa-miR-34b	Not detected	N/A
G03	cfa-miR-34c	Not detected	N/A
G04	cfa-miR-375	4.7691	0.000907
G05	cfa-miR-378	-3.6265	0.112282
G06	cfa-miR-451	-3.9566	0.140974
G07	cfa-miR-499	-1.6478	0.80454
G08	cfa-miR-7	4.1391	0.03274
G09	cfa-miR-9	1.1732	0.494071
G10	cfa-miR-92a	2.5722	0.002914
G11	cfa-miR-93	-10.6306	0.548796
G12	cfa-miR-96	Not detected	N/A
